# Enhanced Third‐Order Nonlinear Optics From In Situ Grown Lead‐Free Cs_2_AgInCl_6_ Double Perovskite Nanocrystals in Chalcogenide Glass

**DOI:** 10.1002/nap2.70120

**Published:** 2026-05-17

**Authors:** Ruonan Chen, Feifei Chen, Changgui Lin, Shixun Dai, Tiefeng Xu

**Affiliations:** ^1^ Laboratory of Infrared Material and Devices The Research Institute of Advanced Technologies, Ningbo University Ningbo China; ^2^ Zhejiang Key Laboratory of Advanced Optical Functional Materials and Devices Ningbo University Ningbo China; ^3^ Engineering Research Center for Advanced Infrared Photoelectric Materials and Devices of Zhejiang Province Ningbo Zhejiang China; ^4^ Ningbo Institute of Oceanography Ningbo China

**Keywords:** chalcogenide glass, lead‐free metal halide double perovskites, nanocomposites, nonlinear optics, optical limiting

## Abstract

Metal halide perovskites exhibit significant potential for optoelectronic applications due to their exceptional linear and nonlinear optical properties. However, the lead toxicity, coupled with the inherent thermal and photochemical instability of conventional lead‐based perovskites, restricts their utility in nonlinear photonic devices that require high‐energy excitation. Lead‐free metal halide double perovskites (MHDP), notably Cs_2_AgInCl_6_, have emerged as environmentally benign alternatives due to their superior structural stability. Nevertheless, systematic investigations of their third‐order nonlinear (TON) optical properties remain quite limited. In this work, we use chalcogenide glass (ChG) with intrinsically high TON to host in situ crystallized Cs_2_AgInCl_6_ nanocrystals, creating a lead‐free nanocomposite material. Using Z‐scan and I‐scan techniques, the synthesized MHDP‐ChG composite was found to exhibit high nonlinear refractive indices, large two‐photon absorption coefficients, and low optical limiting thresholds at near‐infrared wavelengths. This work establishes a novel strategy for lead‐free, high‐TON‐performance MHDP materials and provides critical insights into their applications in nonlinear photonics.

## Graphical abstract

This work successfully achieves the in situ nanocrystallization of lead‐free Cs_2_AgInCl_6_ double perovskite within a chalcogenide glass host. The novel composite demonstrates a remarkable synergy, leading to a strong enhancement in third‐order nonlinear optical properties and a substantially improved optical limiting response, thereby offering a stable and lead‐free candidate for next‐generation nonlinear photonic devices.

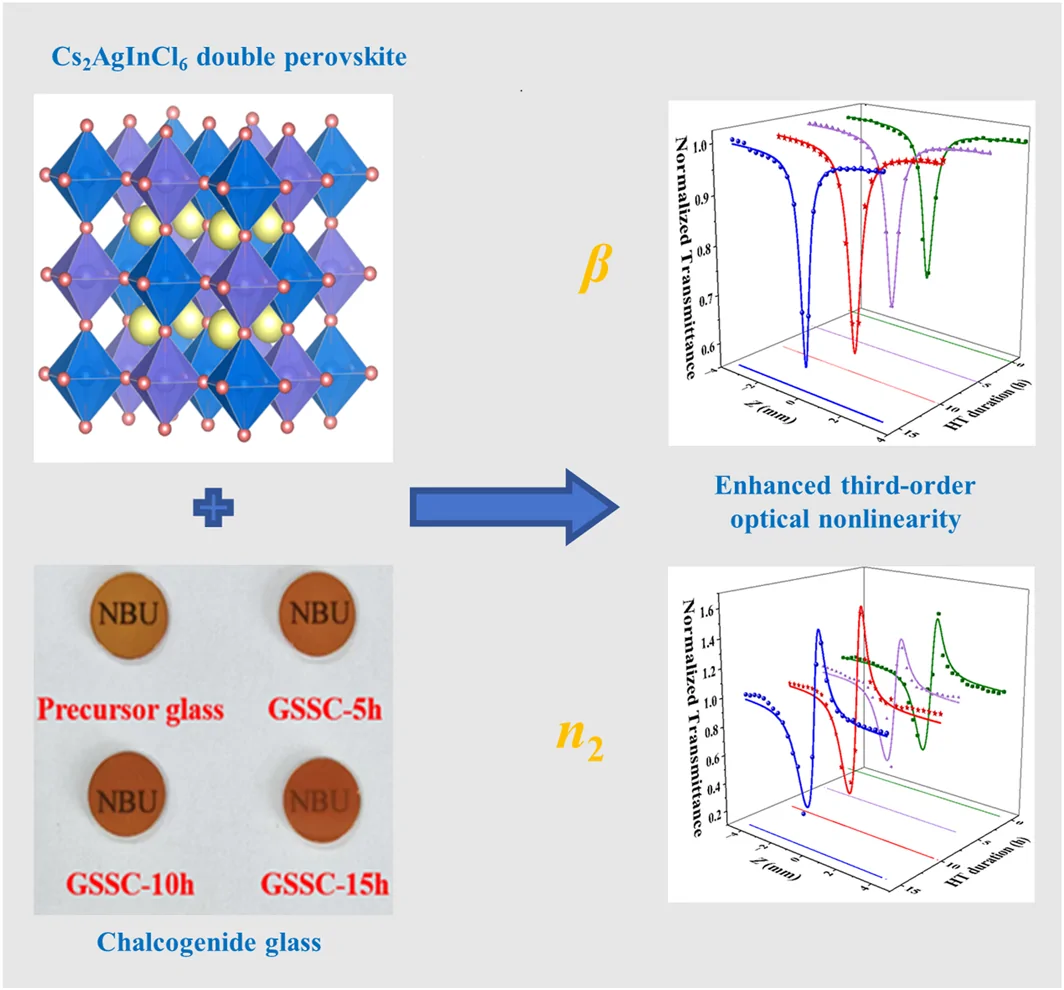

## 1 Introduction

In recent years, low‐dimensional materials, including two‐dimensional materials [1, 2] and perovskite nanocrystals [3, 4], have attracted considerable interest for their unique optical and electronic properties. Among them, metal halide perovskites (MHP) [5, 6] have attracted considerable research interest due to their exceptional nonlinear optical properties, including large two‐photon absorption cross‐sections, pronounced Kerr nonlinearity, and efficient upconversion fluorescence. These attributes—combined with tunable bandgaps, high exciton binding energies, and structural diversity—endow MHP with considerable potential for nonlinear photonic applications such as all‐optical switching, upconversion lasing, and optical limiting [7, 8, 9]. However, lead toxicity and inherent photothermal instability in conventional MHP pose critical limitations. Initial mitigation strategies employed Sn^2+^ or Ge^2+^ substitution for Pb^2+^ [10], yet oxidative degradation under ambient conditions compromised stability; alternative approaches utilizing trivalent cations (e.g., Bi^3+^, Sb^3+^) formed vacancy‐ordered A_3_M_2_X_9_ structures (A = Cs; M = Bi, Sb; X = Cl, Br, I) [11], but their reduced octahedral connectivity and lower symmetry diminish charge mobility, restricting optoelectronic utility [12, 13]. In contrast, charge‐ordered metal halide double perovskites (MHDP) with the general formula A_2_B^(I)^B^(III)^X_6_—where monovalent (B^(I)^) and trivalent (B^(III)^) cations replace two Pb^2+^ ions [14]—retain face‐centered cubic symmetry analogous to conventional MHP while enabling lead‐free fabrication and superior photothermal stability. Moreover, the dual‐cation architecture provides elemental diversity for multidimensional nonlinear optical property modulation [15, 16], with Cs_2_AgInX_6_‐based MHDP exhibiting direct bandgaps, extended carrier lifetimes, and robust structural integrity, making them strong candidates for advanced nonlinear photonic applications [17, 18].

Research on MHDP materials initiated in 2016, focusing on their synthesis, crystallinity, band structure, and optoelectronic properties, with predominant emphasis on photoluminescence behavior [19, 20, 21, 22, 23]. Conversely, investigations of their third‐order optical nonlinear (TON) properties remain quite limited. Initial breakthroughs came in 2021 when Mushtaq et al. observed two‐photon absorption (2PA) coefficients in Cs_2_AgIn_0.9_Bi_0.1_Cl_6_ nanocrystals exceeding those of CsPbBr_3_ by 2–3 orders of magnitude [24]. Subsequent work by Zhang et al. reported a 2PA cross‐section of ≈1906 GM for Cs_2_AgBiBr_6_ nanocrystals—surpassing the values for CdSe quantum dots [25]. Further enhancement was achieved in 2023 through lanthanide doping of Cs_2_AgInCl_6_ nanocrystals [26]. Nevertheless, systematic studies addressing TON dispersion characteristics and nonlinear photonic applications of MHDP remain critically underdeveloped compared to conventional MHP materials.

Chalcogenide glasses (ChGs), recognized as novel amorphous semiconductors [27], constitute a critical platform for nonlinear and infrared photonics due to their exceptional TON properties, broad infrared transmission window, and low phonon energy. Critically, ChG can serve as ideal host matrices for diverse nanocrystals, with nanocrystal incorporation enabling significant modulation and enhancement of glass properties [28]. Prior work [29, 30] confirms the successful synthesis of conventional MHP nanocrystals within ChG matrices, demonstrating excellent compatibility. Notably, MHP‐ChG composites exhibit substantially enhanced TON performance compared to both the bare ChG matrix and discrete MHP crystals. These findings establish that MHDP‐ChG composites [31, 32] could be promising lead‐free nonlinear optical materials with enhanced photothermal stability and superior TON.

Herein, we report the in situ crystallization of lead‐free Cs_2_AgInCl_6_ MHDP nanocrystals within an 83GeS_2_‐17Sb_2_S_3_ (in molar ratio) ChG matrix, which has been demonstrated to exhibit high compatibility with MHP nanocrystals [29], yielding a novel MHDP‐ChG nanocomposite. The TON properties and dispersion characteristics of this composite material were systematically investigated across 710–870 nm via femtosecond Z‐scan [33] and I‐scan techniques. Results demonstrate that MHDP crystallization substantially enhances both the nonlinear refractive index (*n*_2_) and two‐photon absorption coefficient (*β*_2PA_) in the composites within the near‐infrared region while also improving their optical limiting response.

## 2 Experimental

The precursor glass for the MHDP‐ChG composite was formulated with a molar composition of 97(0.83GeS_2_‐0.17Sb_2_S_3_)‐3(0.5CsCl‐0.25InCl_3_‐0.25AgCl). High‐purity raw materials—germanium (5N), antimony (5N), sulfur (5N), cesium chloride (3N), silver chloride (3N), and indium chloride (3N)—were homogenized (15 g total) in a silica ampoule, all operated in a glovebox under an inert atmosphere due to the hygroscopic nature of indium chloride [34]. The ampoule was then evacuated to < 10^−5^ Torr and flame‐sealed. The thermal protocol comprised three sequential steps: (1) melting at 900°C for 12 h in a rocking furnace, (2) rapid quenching into an aqueous NaOH solution, and (3) annealing below the glass transition temperature (*T*_*g*_ = 265°C) for 10 h to relieve residual stresses. Annealed rods were sectioned into 2‐mm‐thick slices, followed by heat treatment at *T*_*g*_ + 30°C for 5, 10, and 15 h, with the resultant samples designated as GSSC‐5h, GSSC‐10h, and GSSC‐15h.

The structure and morphology of Cs_2_AgInCl_6_ MHDP nanocrystals were characterized by X‐ray diffraction (XRD, D2 PHASER, Bruker, Germany) and transmission electron microscopy (TEM, FEI Talos F200x, FEI, USA). Optical transmission and absorption spectra were obtained across visible‐to‐near‐infrared wavelengths using a UV–Vis–NIR spectrophotometer (Lambda 950, PerkinElmer, USA) and Fourier transform infrared spectrometer (Nicolet 381, Nicolet, USA). Steady‐state photoluminescence spectra were recorded on a fluorescence spectrometer (FLS980, Edinburgh Instruments, UK) with a 480‐W xenon lamp excitation source.

The TON properties of MHDP‐ChG composites were characterized by the Z‐scan technique over 710–870 nm. Measurements used a femtosecond laser (Chameleon Ultra II, Coherent, USA) with an 80‐MHz repetition rate, 140 fs pulse width, and ± 3% power stability. Incident power was maintained below 75 mW to prevent sample damage and supercontinuum generation, with triplicate measurements ensuring reliability. Concurrently, optical limiting performance was evaluated via the I‐scan technique across 710–880 nm. Average laser power was modulated from 50 to 160 mW, whereas transmitted energy density was recorded by a high‐precision Si photodiode (S142C, Thorlabs, USA).

All optical characterizations were conducted at ambient temperature (25°C ± 1°C).

## 3 Results and Discussion

### 3.1 Microstructure

Figure 1a presents XRD patterns of the precursor glass and MHDP‐ChG composites under varied heat treatment durations. The precursor glass exhibits a broad amorphous halo without crystalline peaks. In contrast, heat‐treated samples show progressive in situ nanocrystallization: GSSC‐5h and GSSC‐10h develop sharp peaks at 23.997° and 34.194°, with intensity scaling with duration. GSSC‐15h reveals additional peaks at 42.208° and 49.135°, identified as the cubic Cs_2_AgInCl_6_ phase (ICSD 257115; space group Fm3̅*m*). Figure 1b illustrates the corner‐shared 3D network of [AgCl_6_]^5−^ and [InCl_6_]^3−^ octahedra with Cs^+^ in the interstitial sites. The structure features In‐Cl bond lengths (2.568 Å), which are shorter than the Ag‐Cl bond lengths (2.672 Å). Debye–Scherrer analysis [35] of the XRD patterns confirms that the crystallite size increases from 6.1 nm (GSSC‐5h) to 10.7 nm (GSSC‐10h) to 16.7 nm (GSSC‐15h) with extended heat treatment duration.

**FIGURE 1:**
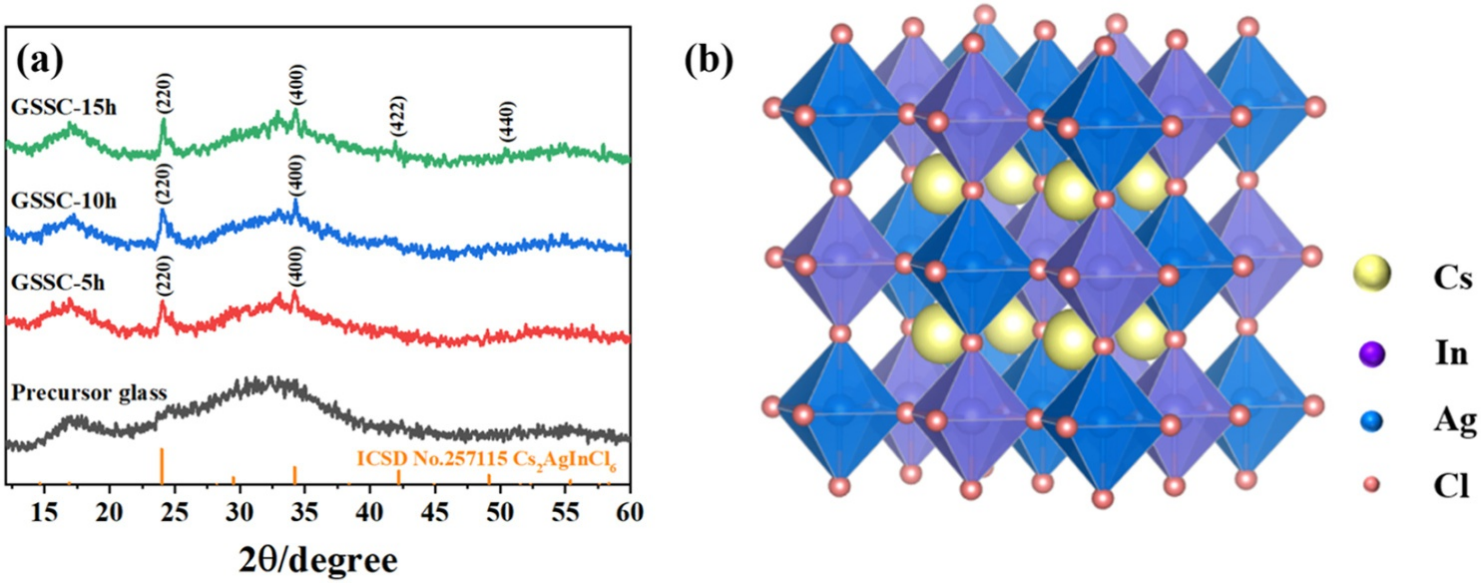
(a) XRD patterns of the samples with different heat treatment durations. (b) Crystal structure of Cs_2_AgInCl_6_.

Figure 2 characterizes the morphology of in situ crystallized Cs_2_AgInCl_6_ in GSSC‐15h via TEM and EDS mapping. Low‐to‐high‐magnification images (Figure 2a,b) reveal uniformly dispersed cubic nanocrystals (average size: 18.1 nm) with sharp boundaries and negligible aggregation. The narrow size distribution histogram (Figure 2a inset) indicates homogeneous crystallization, consistent with the XRD‐derived crystallite size (16.7 nm). High‐resolution TEM (Figure 2c) shows lattice fringes with a d‐spacing of 0.37 nm, indexed to the (220) plane of cubic Cs_2_AgInCl_6_. Selected‐area electron diffraction (SAED, Figure 2d) exhibits concentric rings corresponding to the (220), (400), (422), and (440) planes, confirming complete crystallinity and correlating with the XRD peak assignments in Figure 1a. Figure 2e–l presents the EDS mapping of Cs, Ag, In, and Cl elements in Cs_2_AgInCl_6_, along with Ge, Sb, and S in GeS_2_‐Sb_2_S_3_ ChG. It is clear that all seven elements are distributed uniformly in the composite samples. In detail, the highlighted signals of Cl in Figure 2i show the best match with the grain contours in Figure 2e due to their highest concentration in Cs_2_AgInCl_6_ crystals. The signal of Cs (Figure 2f) in the MHDP crystals is also distinguishable but much weaker compared to that of Cl as a result of its lower concentration (one third that of Cl). For Ag (Figure 2g) and In (Figure 2h), which have concentrations only about half and one sixth those of Cs and Cl, respectively, their X‐ray signals can be detected, but the highlight in crystals is not shown. Meanwhile, the low‐light signal of S (Figure 2l) aligns with the grain contours in Figure 2e, confirming that the Cs_2_AgInCl_6_ nanocrystals are embedded in the sulfur‐based glass matrix.

**FIGURE 2:**
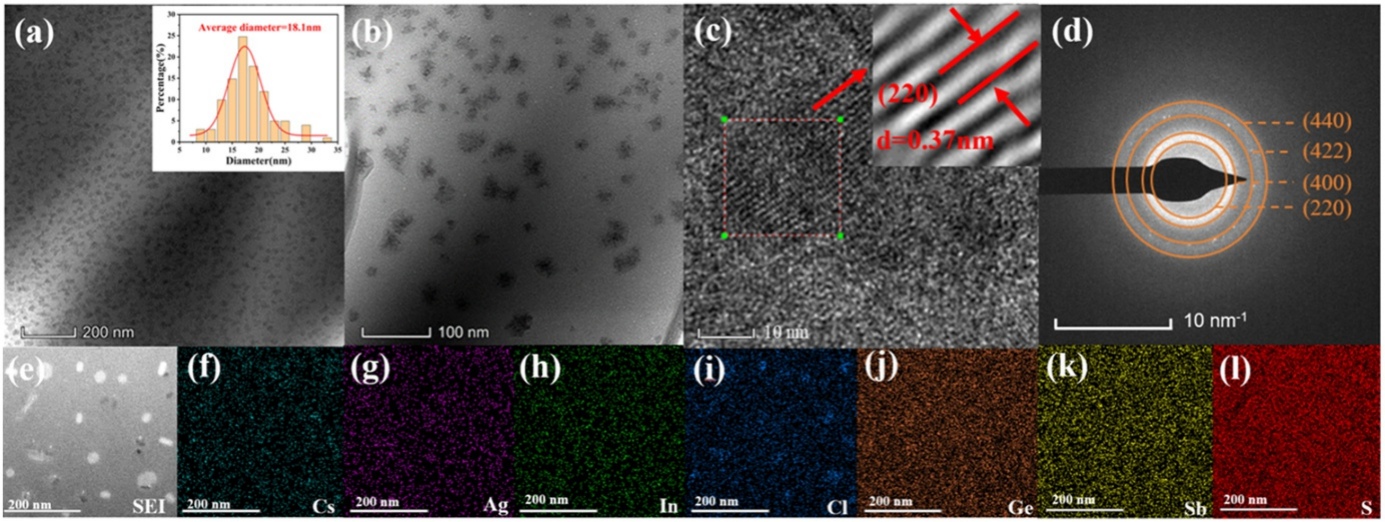
(a) and (b) TEM images of the Cs_2_AgInCl_6_ nanocrystals in GSSC‐15h at different magnifications, with the inset in (a) showing the size distribution histogram, indicating an average diameter of 18.1 nm. (c) High‐resolution transmission electron microscopy image with the inset showing the lattice fringes corresponding to the (220) plane. (d) Selected‐area electron diffraction pattern, which displays diffraction rings indexed to the (220), (400), (422), and (440) crystal planes. (e–l) TEM image of the Cs_2_AgInCl_6_ nanocrystals and the corresponding EDS elemental mappings of Cs, Ag, In, Cl, Ge, Sb, and S, respectively.

### 3.2 Spectral Properties

Figure 3 presents absorption and transmission spectra of the precursor glass and MHDP‐ChG composites. As shown in Figure 3a, extended heat treatment induces progressive redshifting of the short‐wavelength absorption edge due to Cs_2_AgInCl_6_ nanocrystal growth, where the cutoff wavelength (*λ*_cut−off_) is defined at *α* = 100 cm^−1^ [36]. The cutoff wavelength redshifts systematically: 524 nm (precursor glass), 536 nm (GSSC‐5h), 547 nm (GSSC‐10h), and 573 nm (GSSC‐15h). The corresponding optical bandgap energy (*E*_*g*_) can be calculated from these wavelengths using the following equation:

(1) $$E_{g}=\frac{hc}{\lambda_{\text{cut}-\text{off}}},$$

where *h* is Planck's constant and *c* is the speed of light in a vacuum. The calculated *E*_*g*_ decreases systematically: 2.36 eV (precursor glass), 2.31 eV (GSSC‐5h), 2.27 eV (GSSC‐10h), and 2.16 eV (GSSC‐15h), correlating with Cs_2_AgInCl_6_ nanocrystal growth (Figure 3b). This reduction originates from modified electron transitions between the valence and conduction bands induced by nanocrystallization. According to Brunauer–Emmett–Teller theory [37], the specific surface area of nanocrystals scales inversely with size [38, 39]. As Cs_2_AgInCl_6_ nanocrystals grow during extended heat treatment, volume expansion surpasses surface area increase (geometric scaling factor ∝ 1/*r*, where *r* is the mean size of the nanocrystals), reducing the surface area per unit volume. Consequently, diminished interfacial contact area between Cs_2_AgInCl_6_ nanocrystals and the ChG matrix weakens covalent (glass)‐ionic (crystal) bond coupling at heterointerfaces. This interface decoupling suppresses dielectric confinement effects within Cs_2_AgInCl_6_ nanocrystals [40], as evidenced by the progressive *λ*_cut−off_ redshifting in MHDP‐ChG composites, leading to a reduction in their *E*_*g*_ values.

**FIGURE 3:**
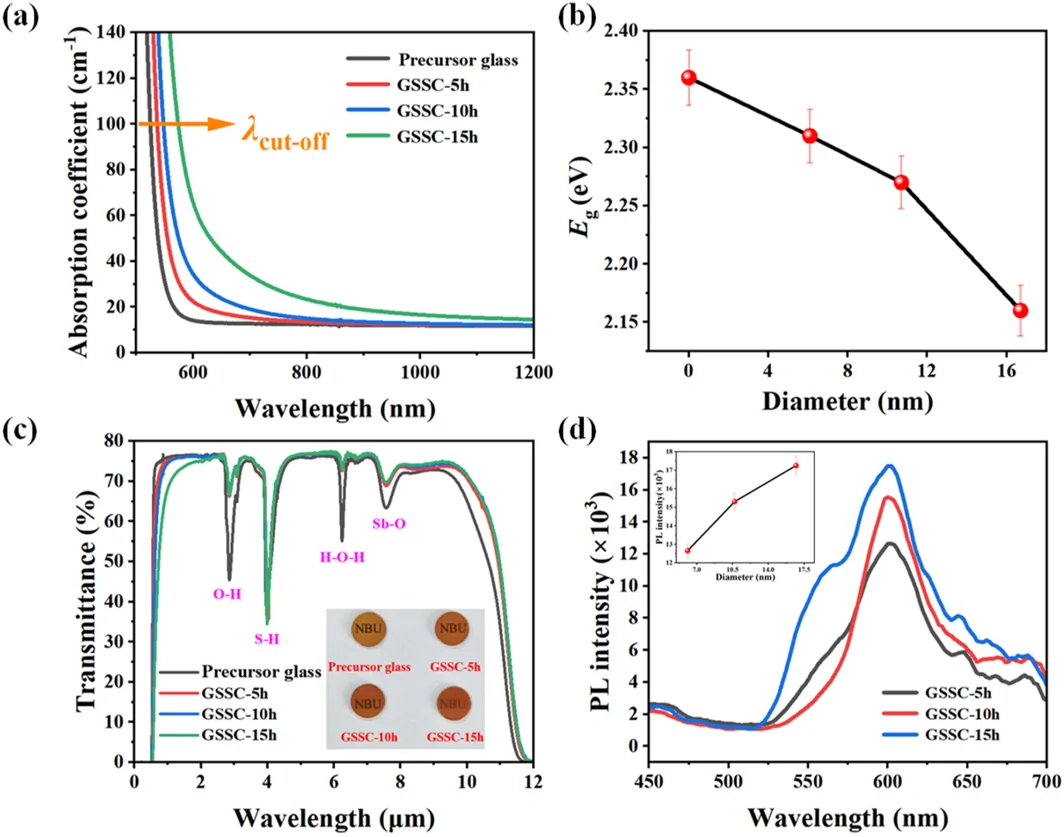
(a) Absorption spectra of the precursor glass and MHDP‐ChG. (b) Relationship between the optical bandgap (*E*_*g*_) and MHDP diameter. (c) Full‐range transmission spectra of the precursor glass and MHDP‐ChG, with the inset showing photographs of the four samples. (d) PL spectra of the MHDP‐ChG under 300 nm excitation, with the inset showing the relationship between MHDP diameter and PL intensity.

Figure 3c shows the optical transmission spectra of the precursor glass and MHDP‐ChG composites from the visible to the mid‐infrared region. It reveals reduced optical transmission across the visible‐to‐near‐infrared regions (0.8–2 μm) upon the in situ crystallization and growth of Cs_2_AgInCl_6_ nanocrystals, which is proved by the evolution in sample appearance (inset in Figure 3c) from bright yellow to dark brown with extended heat treatment. This transmission attenuation arises from enhanced Mie scattering due to the refractive index mismatch between Cs_2_AgInCl_6_ nanocrystals (*n* = 2.0) and the GeS_2_‐Sb_2_S_3_ ChG matrix (*n* = 2.15). At high nanocrystal density, the Tyndall effect scatters light within the matrix, reducing the optical transmittance of MHDP‐ChG in the visible‐to‐near‐infrared region. Conversely, mid‐infrared (2–12 μm) transmission improves significantly with nanocrystal growth, as evidenced by both enhanced transmittance and a redshifted long‐wavelength cutoff edge. Notably, the impurity absorption peaks of O‐H (2.9 μm), H‐O‐H (6.2 μm), and Sb‐O (7.5 μm) are suppressed, whereas the S‐H peak (4 μm) remains unaffected. This indicates that heat treatment not only drives Cs_2_AgInCl_6_ crystallization but also promotes glass network relaxation, reducing oxygen‐related impurities and optimizing long‐wavelength transparency.

Figure 3d presents photoluminescence (PL) spectra of MHDP‐ChG composites under 300 nm xenon‐lamp excitation. All samples exhibit broad emission centered at 600 nm (FWHM ≈ 75–90 nm), consistent with literature reports for Cs_2_AgInCl_6_ MHDP nanocrystals [41], confirming Cs_2_AgInCl_6_ as the dominant emission center in the ChG. This broadband emission originates from Γ‐point conduction‐band‐to‐indirect‐valence‐band transitions involving self‐trapped exciton relaxation and phonon coupling in momentum space [42]. The inset demonstrates monotonic PL intensity enhancement with increasing size of the Cs_2_AgInCl_6_ nanocrystals, verifying intrinsic radiative recombination within the MHDP crystals.

To assess the practical application potential of the MHDP‐ChG composites, their stability under wet conditions and high‐energy laser irradiation was further investigated. In detail, an MHDP‐ChG composite sample (GSSC‐10h) was selected and immersed in deionized water at 85°C for six days (denoted as GSSC‐10h‐Water). Subsequently, this sample was continuously irradiated with a laser at 150 mW/cm^2^ for three hours, yielding the sample denoted as GSSC‐10h‐Laser. Figure 4a compares the XRD patterns of the original GSSC‐10h, GSSC‐10h‐Water, and GSSC‐10h‐Laser. The results show that after water immersion, no significant changes were observed in the intensities of any diffraction peaks. After laser irradiation, the intensity of the main peak (23.997°) remained essentially unchanged, whereas the intensity of the secondary peak (34.194°) decreased only slightly. Furthermore, Figure 4b presents the optical transmittance spectra of the three samples in the visible‐to‐near‐infrared region, revealing no notable differences among them. These results demonstrate that the MHDP‐ChG composite maintains stable optical performance under both hydrothermal conditions and intense laser irradiation, indicating high photothermal and environmental stability.

**FIGURE 4:**
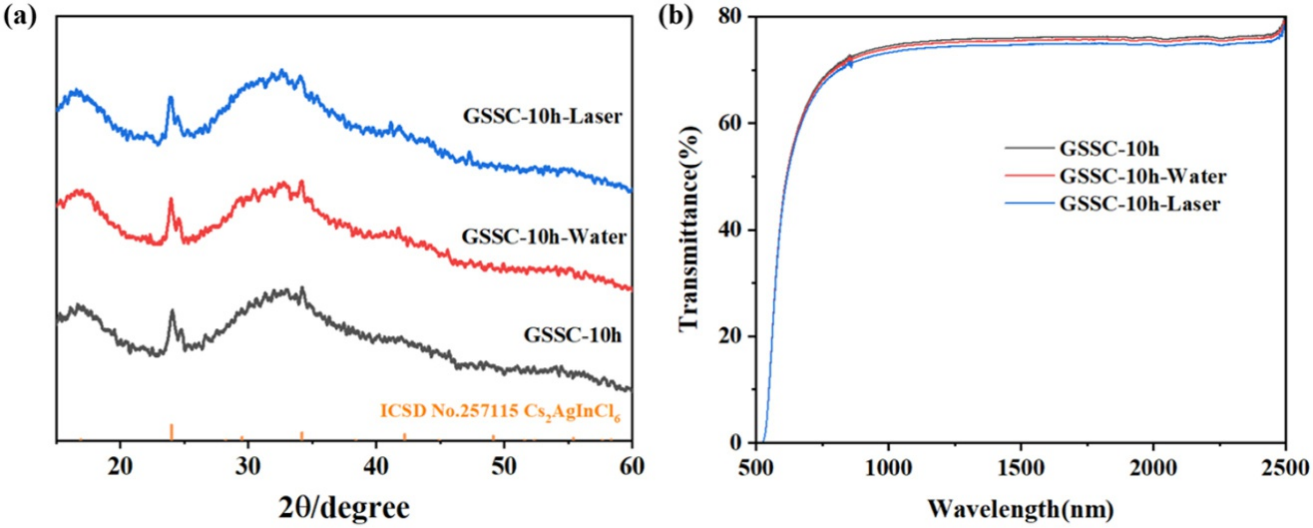
(a) XRD patterns and (b) optical transmittance spectra of the original GSSC‐10h, GSSC‐10h‐Water, and GSSC‐10h‐Laser samples.

### 3.3 Third‐Order Nonlinear Optical (TON) Properties

Using the femtosecond Z‐scan technique, we systematically characterized the TON properties and dispersion behavior of the precursor glass and MHDP‐ChG composites across 710–870 nm at 20 nm spectral intervals. Figure 5 presents representative open‐aperture (OA) and closed‐aperture (CA) Z‐scan traces, which reflect their nonlinear absorption and nonlinear refraction behaviors.

**FIGURE 5:**
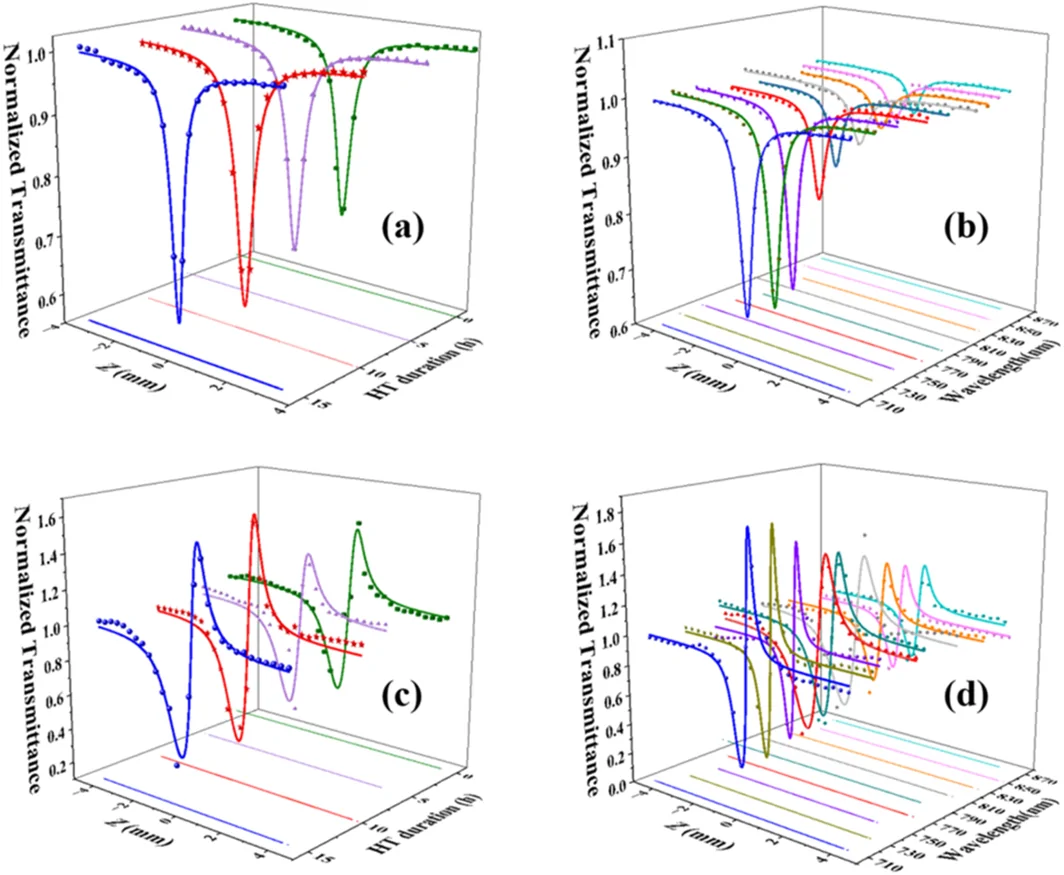
(a) OA and (c) CA Z‐scan curves of the precursor glass and MHDP‐ChG at 710 nm. (b) OA and (d) CA Z‐scan curves of GSSC‐15h in the 710–870‐nm range. The data points represent the experimental results, and the solid lines represent the theoretical fitting.

Figure 5a shows valley‐shaped OA profiles at the focus (*Z* = 0) for all samples, indicating reverse saturable absorption (RSA). In general, RSA in semiconductor materials stems from either multiphoton absorption (MPA) or excited‐state absorption (ESA). To resolve the dominant mechanism in MHDP‐ChG, we measured photoluminescence under 980 nm excitation (Figure 6a), observing a broad 500–650‐nm emission peaking at 600 nm—identical to the 300‐nm single‐photon‐excited PL (Figure 2d). Notably, the sub‐bandgap energy of 980 nm photons (1.27 eV) is below the bandgap energy of the MHDP‐ChG composites (*E*_*g*_ = 2.16–2.31 eV). The negligible linear absorption confirms that the observed emission arises from anti‐Stokes upconversion [43]. Power‐dependent fluorescence intensity for GSSC‐15h (Figure 6b inset) exhibits a log‐log slope of 1.9, demonstrating quadratic dependence characteristic of dominant two‐photon absorption (2PA) under near‐infrared excitation.

**FIGURE 6:**
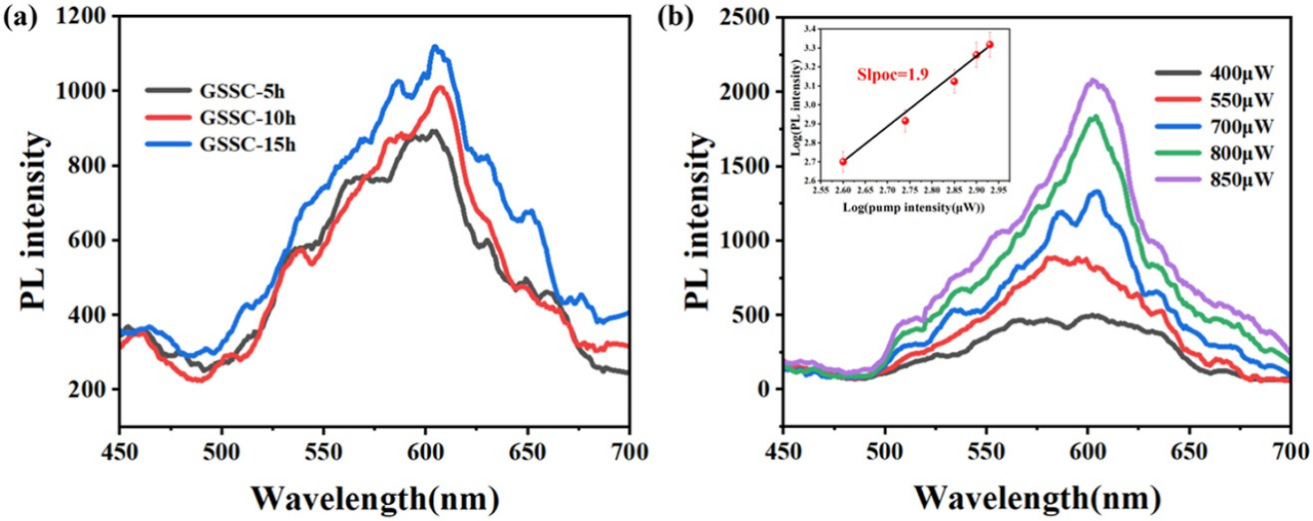
(a) Photoluminescence (PL) spectra of the MHDP‐ChG under 980 nm laser excitation. (b) PL spectra of GSSC‐15h under 980 nm laser excitation (power ranging from 400 to 850 μW). The inset shows the logarithmic plot of PL intensity versus excitation power and its linear fit.

Normally, the 2PA process in semiconductors originates from polarization of atomic outer electron clouds, where ground‐state electrons simultaneously absorb two photons to reach excited states. Its intensity correlates strongly with normalized photon energy (*E*_*p*_/*E*_*g*_), defined as the ratio of incident photon energy (*E*_*p*_) to bandgap energy (*E*_*g*_). For the precursor glass and MHDP‐ChG composites (*E*_*g*_ = 2.16–2.36 eV) under 710–870 nm excitation (*E*_*p*_ = 1.43–1.75 eV), *E*_*p*_/*E*_*g*_ ranges from 0.60 to 0.81 within the canonical 2PA window (0.5 < *E*_*p*_/*E*_*g*_ < 1), confirming 2PA as the dominant nonlinear absorption mechanism in OA Z‐scan measurements as well. Figure 5a demonstrates deepening OA valleys with prolonged heat treatment, indicating enhanced nonlinear phase shift and 2PA intensity. This enhancement stems from Cs_2_AgInCl_6_ nanocrystals with intrinsically large multiphoton absorption cross‐sections [25]. Figure 5b reveals 2PA dispersion in GSSC‐15h: Valley depth decreases progressively from 710 to 870 nm, showing inverse wavelength dependence. Fitting OA traces with the standard 2PA model [44] (Supporting Information S1: Equation S1) yields *β*_2PA_ values (Figure 7a; full data in Supporting Information S1: Table S1). At 710 nm, *β*_2PA_ reaches 1.23 × 10^−10^ m/W for GSSC‐15h—more than two times that of the precursor glass. Even at 870 nm, GSSC‐15h maintains 0.61 × 10^−11^ m/W, still more than two times the precursor glass value, demonstrating that the growth trend exhibits full‐band uniformity.

**FIGURE 7:**
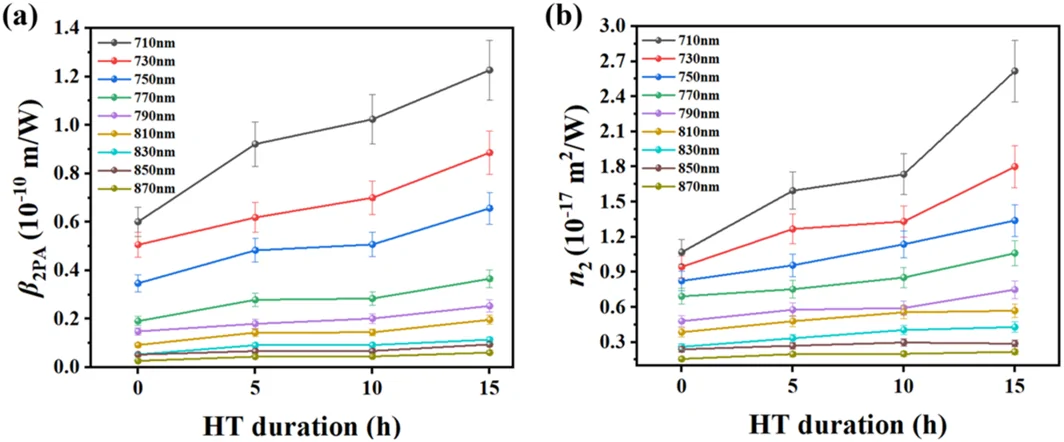
Variation of *β*_2PA_ (a) and *n*_2_ (b) with heat treatment (HT) duration.

Figure 5c presents CA Z‐scan traces at 710 nm, exhibiting valley‐peak configurations indicative of self‐focusing behavior [45]. This signature—a prefocal transmission valley followed by a postfocal peak—confirms a positive nonlinear refractive index (*n*_2_ > 0). Mirroring the OA trends, the valley‐peak differential amplifies with Cs_2_AgInCl_6_ nanocrystal growth, signaling enhanced *n*_2_. Figure 5d reveals wavelength‐dependent CA scans for GSSC‐15h, where valley‐peak contrast diminishes with increasing excitation wavelength, demonstrating normal dispersion consistent with the OA behavior. Fitting CA curves via the Kwak model (Supporting Information S1: Equation S2) [46] yields *n*_2_ values (full data in Supporting Information S1: Table S2). Figure 7b confirms *n*_2_ enhancement with heat treatment duration, with GSSC‐15h achieving peak *n*_2_ values across 710–870 nm. The observed *n*_2_ enhancement can be partly attributed to amplification of the local field resulting from the presence of Cs_2_AgInCl_6_ nanocrystals in the GeS_2_‐Sb_2_S_3_ glass. According to the Maxwell Garnett effective medium theory for spherical nanoparticles embedded in a dielectric matrix, the local field enhancement factor of the MHDP‐ChG is given by the following equation [47]:

(2) $$f_{\text{loc}}=\frac{3\varepsilon_{m}}{\varepsilon_{p}+2\varepsilon_{m}},$$

where *ε*_*p*_ = *n*_*p*_^2^ and *ε*_*m*_ = *n*_*m*_^2^ are the dielectric constants of the nanoparticle and matrix, respectively. Using *n*_*p*_ ≈ 2.0 for Cs_2_AgInCl_6_ nanocrystals and *n*_*m*_ ≈2.15 for GeS_2_‐Sb_2_S_3_ glass, the local field enhancement factor was calculated to be 1.047. Because the third‐order nonlinear susceptibility *χ* ^(3)^ scales as |*f*_loc_|^4^ [48], the expected enhancement factor for the *n*_2_ value of the MHDP‐ChG is 1.2. In addition, the dual enhancement of 2PA and nonlinear refraction (NLR) indicates the occurrence of two‐photon resonance effects under 2PA conditions (0.5 < *E*_*p*_/*E*_*g*_ < 1). Such resonance elevates photon–electron interactions, amplifying the broadband NLR response of the MHDP‐ChG composites.

Table 1 benchmarks the TON parameters of the MHDP‐ChG composite against those of the host GeS_2_‐Sb_2_S_3_ glass and other inorganic MHP/MHDP materials reported in recent studies [24, 25, 26, 49, 50, 51, 52, 53, 54]. Firstly, the *β* and *n*_2_ values of Cs_2_AgInCl_6_ MHDP‐ChG remarkably surpass those of the GeS_2_‐Sb_2_S_3_ host glass [49], indicating that the incorporation of Cs_2_AgInCl_6_ nanocrystals enhances the third‐order nonlinearity of the chalcogenide glass. Secondly, compared to the *n*_2_ value of pristine Cs_2_AgInCl_6_ nanocrystals (0.9 × 10^−19^ m^2^/W, dispersed in n‐hexane) [26], those of the MHDP‐ChG composite (0.2 to 2.6 × 10^−17^ m^2^/W) are enhanced by at least two orders of magnitude, highlighting the intrinsically high TON performance of the host ChG in providing a high baseline for the nonlinear optical performance of the nanocomposites. Thirdly, the *β* and *n*_2_ values of the Cs_2_AgInCl_6_ MHDP‐ChG composite are one to three orders of magnitude higher than those of CsPbCl_3_ nanocrystals in n‐hexane colloids [50], Cs_2_NaBiI_6_ thin films [51], and Cs_2_AgBiBr_6_ thin films [25], fully demonstrating the synergistic effect of the MHDP‐ChG integration strategy on enhancing the nonlinear optical response. In addition, it should be noted that although the nonlinear optical parameters of the present MHDP‐ChG are wavelength‐dependent, the *β* values of sample GSSC‐15h (at 790 and 810 nm; see Supporting Information S1: Table S1) are still higher than those of CsPbBr_3_ thin films [52] (at 800 nm). Furthermore, the MHDP‐ChG achieves *β* and *n*_2_ values comparable to advanced benchmarks, including CsSnCl_3_ MHP‐ChG [32], composition‐optimized Cs_2_AgIn_0.9_Bi_0.1_Cl_6_ thin films [24], and even organic‐inorganic hybrid MHPs (such as MAPbBr_3_ thin films [53] and (PEA)_2_PbI_4_ thin films [54]), indicating substantial optimization potential. Targeted engineering of B‐site cations and halide substitutions in the MHDP nanocrystals could unlock even higher TON performance.

**TABLE 1:** Comparison of the TON parameters of the MHDP‐ChG against GeS_2_‐Sb_2_S_3_ and other reported inorganic MHP/MHDP materials in recent studies.

Material	Structure	*λ*	*β* (× 10^−11^ m/W)	*n*_2_ (× 10^−17^ m^2^/W)	Ref.
Cs_2_AgInCl_6_/ChG	Nanocrystals	710–870 nm	0.5–12.3	0.2–2.6	This work
GeS_2_‐Sb_2_S_3_ ChG	Host glass	800 nm	0.8–1.7	0.4–0.7	[49]
Cs_2_AgInCl_6_/hexane	Nanocrystals	800 nm	—	0.9 × 10^−2^	[26]
CsPbCl_3_/hexane	Quantum dots	787 nm	1.4 × 10^−2^	—	[50]
Cs_2_NaBiI_6_	Film	1030 nm	6.2 × 10^−3^	1.1 × 10^−1^	[51]
Cs_2_AgBiBr_6_	Film	—	14.9 × 10^−2^	—	[25]
CsPbBr_3_	Film	800 nm	1.8	1.8	[52]
CsSnCl_3_/ChG	Nanocrystals	750–900 nm	1.7–23.9	0.5–2.9	[32]
Cs_2_AgIn_0.9_Bi_0.1_Cl_6_	Film	800 nm	21.9	2.6	[24]
MAPbBr_3_	Film	1060 nm	53.0	3.8	[53]
(PEA)_2_PbI_4_	Film	800 nm	12.6	—	[54]

Figure 8 depicts *β*_2PA_ and *n*_2_ versus *E*_*p*_/*E*_*g*_ for all samples, revealing a positive correlation between the TON parameters and normalized photon energy. For *β*_2PA_ dispersion, this trend aligns with the relativistic effect‐induced two‐step rise in the 2PA spectrum of Cs_2_AgInCl_6_ nanocrystals, where spin–orbit coupling in heavy atoms (Ag, In) splits the conduction band into heavy‐ and light‐electron states [55]. The resultant secondary bandgap drives accelerated *β*_2PA_ enhancement at higher *E*_*p*_/*E*_*g*_, as noted in Figure 8a. For *n*_2_ dispersion in Figure 8b, the monotonic increase is primarily attributed to free‐carrier contributions arising from one‐photon and two‐photon reabsorption in the tail region of the ChG. In parallel, highly crystalline GSSC‐15h exhibits a pronounced spectral deviation toward *E*_*p*_/*E*_*g*_ = 1. The host ChG and the MHDP crystals are indirect and direct bandgap semiconductors, respectively. Consequently, the combination of these two different band structures causes the energy bands of the composites to distort, fold, and split at the interface, leading to a loss of long‐range order in the resultant band structure. Additionally, the size distribution and spatial disorder of the Cs_2_AgInCl_6_ nanocrystals fragment their energy bands as well. These effects reduce long‐range order, shifting dispersion curves toward *E*_*p*_/*E*_*g*_ = 1, which is consistent with the nonlinear dispersion trend of direct gap semiconductors [56] versus indirect gap semiconductors [57].

**FIGURE 8:**
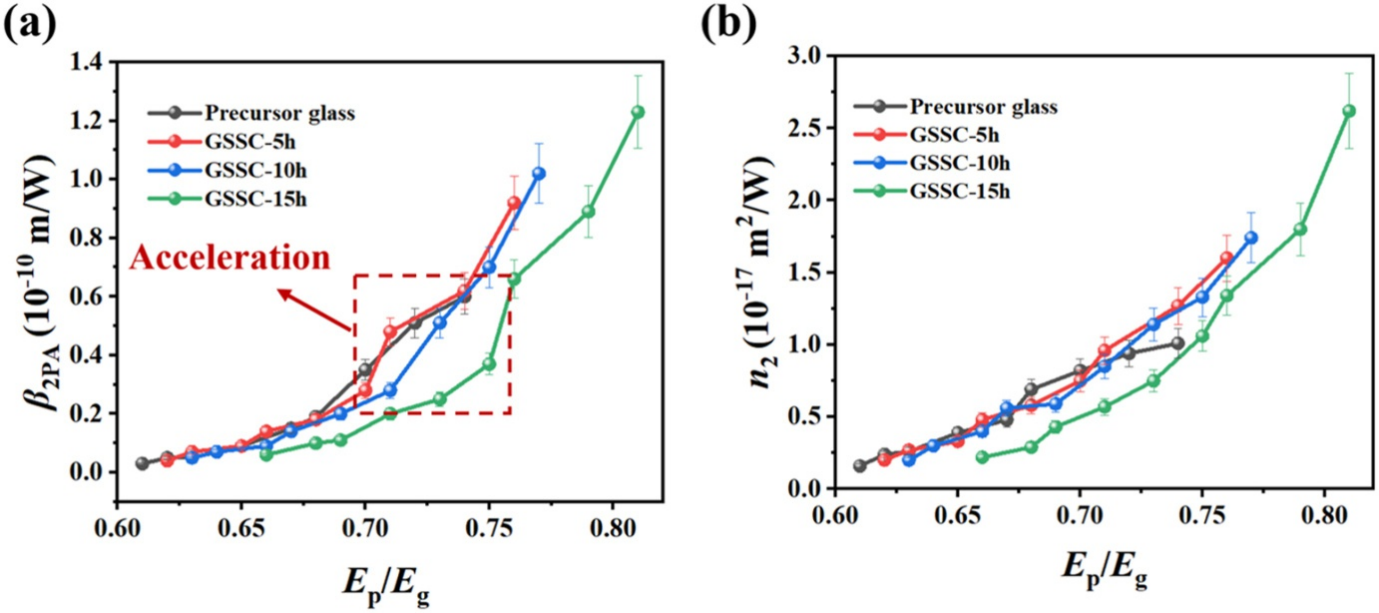
(a) Relationship between *β*_2PA_ and *E*_*p*_/*E*_*g*_ for the precursor glass and MHDP‐ChG. (b) Relationship between *n*_2_ and *E*_*p*_/*E*_*g*_ for the precursor glass and MHDP‐ChG.

The Z‐scan results demonstrate that MHDP‐ChG composites exhibit high *n*_2_ and *β*_2PA_ values with normal dispersion characteristics. To evaluate the trade‐off between *n*_2_ and *β* for photonic device applicability, we calculated the figure of merit (FOM) using the following equation:

(3) $$\text{FOM}=\frac{n_{2}}{\beta_{2\text{PA}}\lambda },$$

where *λ* denotes the excitation wavelength. Materials with a FOM > 1 are typically suitable for *n*_2_‐based applications such as all‐optical switching and supercontinuum generation, whereas those exhibiting FOM < 1 are more appropriate for multiphoton absorption‐driven applications, including optical limiting and upconversion lasers (full data in Supporting Information S1: Table S3). As shown in Figure 9a, the FOM dispersion curve for MHDP‐ChG composites remains below 1 across the entire spectral range. This confirms that the nonlinear optical effects are dominated by 2PA, rendering the material particularly suitable for nonlinear absorption‐based photonic devices. The primary reason for the stronger 2PA than NLR in MHDP‐ChG composites originates from the direct bandgap characteristic of Cs_2_AgInCl_6_ nanocrystals, which facilitates two‐photon transition channels and confers a high intrinsic 2PA cross‐section. Consequently, Cs_2_AgInCl_6_ nanocrystallization enhances the probability of valence‐to‐conduction‐band electron transitions via 2PA, resulting in enhanced *β*_2PA_ for the MHDP‐ChG composites. Figure 9b further correlates the average FOM with the crystallinity of the Cs_2_AgInCl_6_ nanocrystals (represented by their average diameter). All samples exhibit average FOM values below 0.5, with the highest‐crystallinity specimen (GSSC‐15h) demonstrating the lowest value and minimal deviation, indicating optimal 2PA performance.

**FIGURE 9:**
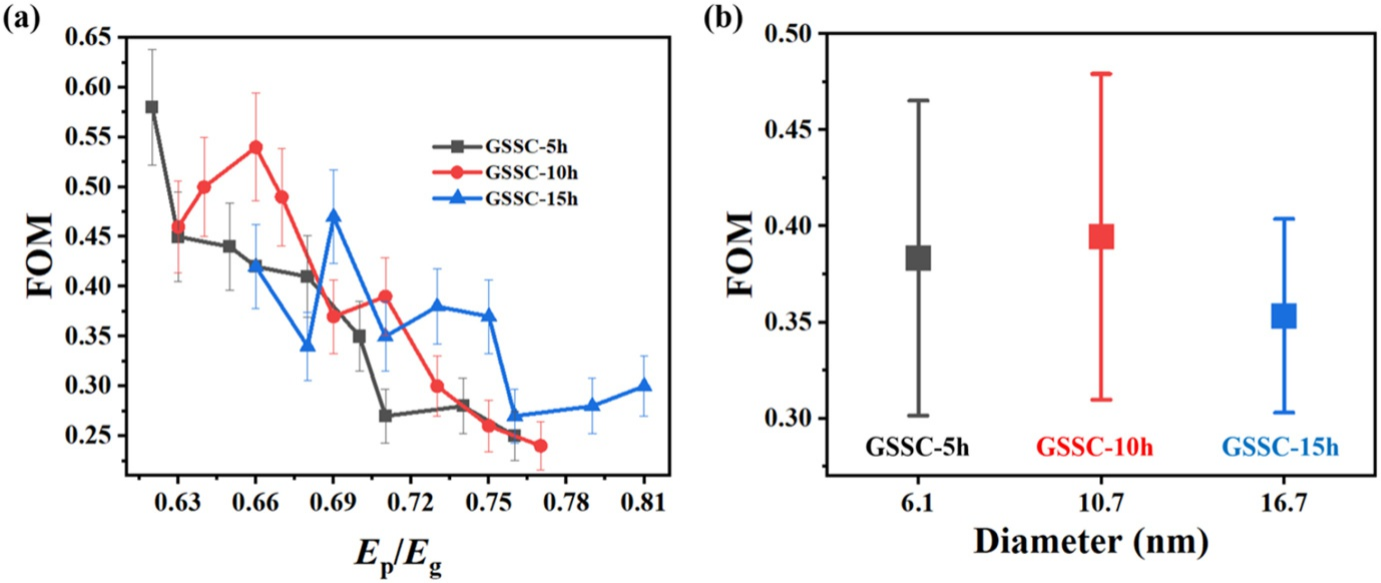
(a) Dispersion curves of the FOM for the MHDP‐ChG. (b) Relationship between the average FOM and Cs_2_AgInCl_6_ crystal diameter.

### 3.4 Optical Limiting Performance

To further characterize the enhanced 2PA properties of MHDP‐ChG composites, comparative I‐scan analysis of optical limiting (OL) performance was conducted between the precursor glass and GSSC‐15h (lowest average FOM). The optical limiting threshold (OLT), defined as the incident energy density at which normalized transmittance decreases to 0.707 [58], was evaluated across 710–880 nm (full data in Supporting Information S1: Table S4). As shown in Figure 10a, 15 h heat treatment reduced the OLT at 710 nm by 76.7%, from 345.0 μJ/cm^2^ (precursor glass) to 80.5 μJ/cm^2^ (GSSC‐15h), confirming significant OL enhancement via Cs_2_AgInCl_6_ nanocrystallization. Figure 10b demonstrates wavelength‐dependent OLT evolution for GSSC‐15h, revealing a strong negative correlation with excitation wavelength: OLT increased from 80.5 μJ/cm^2^ (710 nm) to 381.6 μJ/cm^2^ (880 nm). This trend aligns with the positive dispersion of *β*_2PA_ values in the Z‐scan results, establishing consistent structure–property relationships for nonlinear absorption.

**FIGURE 10:**
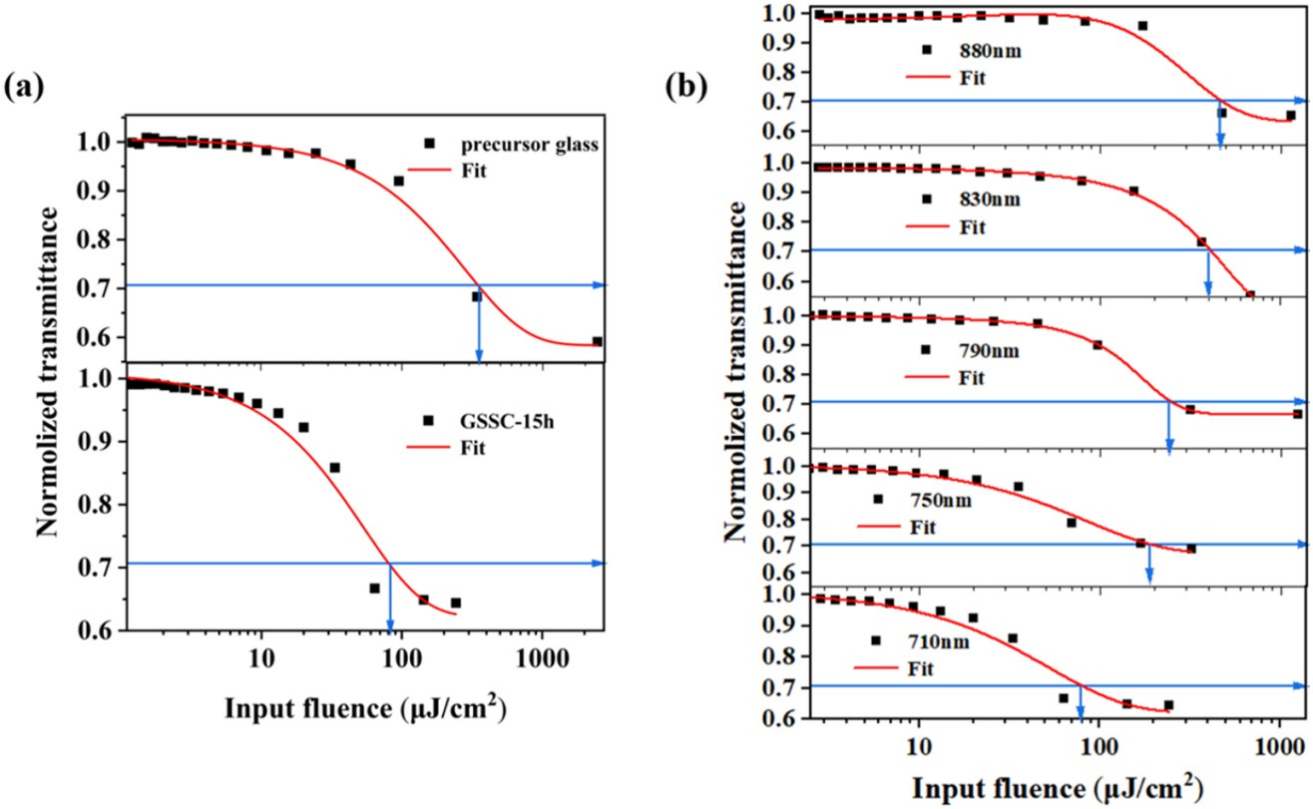
(a) I‐scan curves of the precursor glass and GSSC‐15h at 710 nm. (b) I‐scan curves of GSSC‐15h at 710, 750, 790, 830, and 880 nm. The data points in the figure represent experimentally obtained results, and the solid lines represent the theoretical fitting.

Table 2 compares the OLT of the MHDP‐ChG composite developed in this work with those of other reported MHP/MHDP materials [32, 58, 59, 60, 61, 62, 63, 64, 65]. The results demonstrate that the OLT for MHDP‐ChG is on the order of μJ/cm^2^. This value is comparable to those of other MHP‐ChG composites reported in our previous studies [32, 58], and it is two orders of magnitude lower than those measured for Mn‐doped Cs_2_AgInCl_6_ MHDP materials in anhydrous n‐hexane [59] and CsPbI_2_Br in toluene [61]. Furthermore, it is five orders of magnitude lower than the OLT values of conventional MHPs, including Cs_3_Sb_2_Br_9_ in ethyl acetate [60], Cs_3_Cu_2_Br_5_ [63], MAPbBr_3_ [64], and CsPbBr_3_ in organic glass (PMMA) [62], and CsPbBr_3_ in inorganic glass (SiO_2_) [65]. The markedly suppressed OLT confirms the superior OL performance of MHDP‐ChG over MHP systems in traditional matrices—including organic solutions, polymer glasses, and oxide inorganic glasses.

**TABLE 2:** Comparison of the OLT parameters of the present MHDP‐ChG against the OLT parameters of other MHP/MHDP materials reported in recent studies.

Material	Excitation wavelength and other laser parameters	Original OLT (μJ/cm^2^)	Normalized OLT_100*fs*_ (μJ/cm^2^)	Ref.
Cs_2_AgInCl_6_/ChG	710–880 nm, 140 fs	80.5–381.6	57.5–272.6	This work
CsSnCl_3_/ChG	700–850 nm, 140 fs	45.2–396.7	32.3–283.4	[32]
CsPbBr_3_/ChG	800–950 nm, 140 fs	32.4–192.1	23.1–137.2	[58]
Mn‐doped Cs_2_AgInCl_6_/anhydrous n‐hexane	800 nm, 70 fs	18.4 × 10^2^	2628.6	[59]
Cs_3_Sb_2_Br_9_ quantum dots/ethyl acetate	532 nm, 7 ns	20 × 10^5^	28.6	[60]
CsPbI_2_Br/toluene	800 nm, 60 fs	6.2 × 10^2^	1033.3	[61]
CsPbBr_3_/PMMA	532 nm, 6 ns	64 × 10^5^	106.7	[62]
Cs_3_Cu_2_Br_5_/PMMA	532 nm, 6 ns	38 × 10^5^	63.3	[63]
MAPbBr_3_/PMMA	532 nm, 6 ns	3.1 × 10^5^	5.2	[64]
CsPbBr_3_ quantum dots/SiO_2_	532 nm, 7 ns	16.8 × 10^5^	24.0	[65]

Considering that OLT is highly pulse‐width dependent, a fairer comparison of these OL materials was conducted via a linear normalization method to unify their OLT values to a pulse width of 100 fs. Based on the linear dependence of the OLT on pulse duration in 2PA‐dominated optical limiting processes [66], the normalization was performed using the following formula:

(4) $${\text{OLT}}_{100fs}={\text{OLT}}_{\text{measured}}\times \frac{100fs}{\tau_{\text{meas}}},$$

where *τ*_meas_ is the laser pulse width used in the original experiment. As given in Table 2, the normalized OLT_100*fs*_ value for the Cs_2_AgInCl_6_ MHDP‐ChG composite is between 57.5 and 272.6 μJ/cm^2^ over the 710–880‐nm range, still comparable to those of the MHP‐ChG measured at identical *τ*_meas_ values. For those tested at shorter *τ*_meas_ values, the normalized OLT_100*fs*_ of the MHDP‐ChG composite is several times lower than those of Mn‐doped Cs_2_AgInCl_6_ MHDP in anhydrous n‐hexane [59] and CsPbI_2_Br in toluene [61], which verifies a synergistic 2PA mechanism between the ChG matrix and the MHDP: The ChG matrix provides a highly optically nonlinear baseline, whereas the larger intrinsic multiphoton absorption cross‐section of Cs_2_AgInCl_6_ nanocrystals, combined with local field enhancement, contributes to an improved OL performance. However, the normalized OLT_100*fs*_ of the MHDP‐ChG composite is remarkably higher than those of Cs_3_Sb_2_Br_9_ in ethyl acetate [60], Cs_3_Cu_2_Br_5_ [63], MAPbBr_3_ [64], and CsPbBr_3_ in organic glass (PMMA) [62], and CsPbBr_3_ quantum dots in inorganic glass (SiO_2_) [65], which were obtianed at a visible wavelength (532 nm) using nanosecond *τ*_meas_ values. This is related to the normal dispersion characteristic of the nonlinear absorption behavior of MHP/MHDP materials, as well as the excited‐state absorption or thermal effects generated under nanosecond‐pulse laser irradiation [67].

## 4 Conclusion

This study presents the first report on the in situ nanocrystallization of lead‐free Cs_2_AgInCl_6_ MHDP nanocrystals in ChG for the successful synthesis of a novel MHDP‐ChG composite, enabling the systematic characterization of its TON properties from 710 to 870 nm via femtosecond Z‐scan and I‐scan techniques. Owing to the large multiphoton absorption cross‐section of Cs_2_AgInCl_6_ nanocrystals and the inherent high TON of the ChG matrix, the composites achieve maximum nonlinear *n*_2_ and *β*_2PA_ values of 2.62 × 10^−17^ m^2^/W and 1.23 × 10^−10^ m/W, respectively. These values are more than twice those of the precursor glass and exceed the values of other MHP/MHDP materials in traditional matrices, such as organic solutions, polymer glasses, and oxide glasses.

The MHDP‐ChG composites demonstrate a low OLT of 80.5 μJ/cm^2^, below one quarter of the value of the precursor glass at an identical wavelength, while also substantially outperforming other MHP/MHDP materials in recent reports. This work demonstrates the potential of lead‐free MHDP‐ChG composites as high‐performance nonlinear optical materials and as a promising platform for lead‐free photonic devices. The combined advantages of superior nonlinearity and stability suggest broad applications in optical limiting and all‐optical photonic technologies.

Future work will focus on exploring different ChGs (e.g., selenide or telluride glasses) that exhibit larger optical nonlinearity so as to provide a higher baseline for the nonlinear optical performance of the nanocomposites. Further optimization of these properties could be achieved through targeted B‐site cation doping (e.g., Bi, Na) and halide substitution (Br, I) in the MHDP nanocrystals.

## Author Contributions

**Ruonan Chen:** conceptualization, methodology, investigation, data curation, formal analysis, writing – original draft. **Feifei Chen:** supervision, project administration, funding acquisition, writing – review and editing, formal analysis, validation. **Changgui Lin:** validation, supervision, resources. **Shixun Dai:** supervision, project administration, resources. **Tiefeng Xu:** supervision.

## Funding

This work was financially supported by the Natural Science Foundation of Zhejiang Province (LZ25F050009), the Natural Science Foundation of Ningbo (2023J016), and the K.C. Wong Magna Fund in Ningbo University.

## Conflicts of Interest

The authors declare no conflicts of interest.

## Supporting information


supporting Information S1

## Data Availability

Data are available from the corresponding author upon reasonable request.
