# Supplementary material for: Enhanced Third‐Order Nonlinear Optics From In Situ Grown Lead‐Free Cs2AgInCl6 Double Perovskite Nanocrystals in Chalcogenide Glass
Source: Nanophotonics. 2026 May 17;15(10):e70120. doi: 10.1002/nap2.70120 (PMC13642478; doi:10.1002/nap2.70120)
Supplement: Supplementary file 1 [file NAP2-15-e70120-s001.docx]

**SUPPLEMENTARY MATERIAL**

1. **Fitting of Z-scan Data**

The open-aperture (OA) Z-scan traces were fitted according to the established two-photon absorption (2PA) model using the following expression:

$T=\frac{\ln[1+q{}_{0}(z)]}{q{}_{0}(z)}$ (Eq.S1)

Where,$q_{0}(\text{z})=q_{0}(0,z)=q_{00}/\left(1+x^{2}\right)$ , $q_{00}=\beta I_{0}L_{\text{eff}}$, Here, *I*_0_ is the peak intensity of the input beam at the focus, *x* = *z*/*z*_0_, *z*_0_ = *kw*_0_^2^/2 is the diffraction length of the beam, *k* = 2π/*λ* is the wavenumber, and *w*_0_ is the beam waist radius of the laser. The effective path length of the sample is given by$L_{\text{eff}}=[1-\exp(-\alpha L)]/\alpha$, where L is the sample thickness and α is the linear absorption coefficient.

According to Kwak's method, the closed-aperture (CA) Z-scan traces were fitted using the following expression:

$T(z)=\frac{1}{1-\frac{(4x+\eta)}{\left(1+q_{0}\right)\left(1+x^{2}\right)^{2}}\Delta\Phi+\frac{4+\eta^{2}}{\left(1+q_{0}\right)\left(1+x^{2}\right)^{3}}\Delta\Phi^{2}}$ （Eq.S2）

Where Δ*Φ* is the on-axis phase shift at the focus, given by Δ*Φ* = *k*Δ*n*(0, 0). Here, Δ *n*(0, 0) = *γI*_0_ , and *γ* = 40π*n*_2_/c*n*_0_，The parameter *η* = *β*/2*kγ* is a coupling factor, representing the ratio of the imaginary part to the real part of the complex nonlinear phase shift.

2. **Third-order nonlinear (TON) parameters**

**Table S1.** Two-photon absorption coefficients (*β*) of the MHDP-ChG samples at wavelengths ranging from 710 nm to 870 nm.

| *β*±10%(10^-11^m/W) | | | | | | | | | |
| --- | --- | --- | --- | --- | --- | --- | --- | --- | --- |
| S.No | 710nm | 730nm | 750nm | 770nm | 790nm | 810nm | 830nm | 850nm | 870nm |
| precursor glass | 6.01 | 5.06 | 3.47 | 1.91 | 1.49 | 0.93 | 0.54 | 0.53 | 0.27 |
| GSSC-5 h | 9.22 | 6.19 | 4.83 | 2.79 | 1.80 | 1.44 | 0.92 | 0.68 | 0.45 |
| GSSC-10 h | 10.24 | 7.00 | 5.07 | 2.84 | 2.02 | 1.45 | 0.93 | 0.68 | 0.46 |
| GSSC-15 h | 12.27 | 8.86 | 6.57 | 3.66 | 2.54 | 1.97 | 1.15 | 0.95 | 0.61 |

**Table S2.** Nonlinear refractive indices (*n*_2_) of the MHDP-ChG samples at wavelengths ranging from 710 nm to 870 nm.

| *n_2_*±10%(10^-17^m^2^/W) | | | | | | | | | |
| --- | --- | --- | --- | --- | --- | --- | --- | --- | --- |
| S.No | 710nm | 730nm | 750nm | 770nm | 790nm | 810nm | 830nm | 850nm | 870nm |
| precursor glass | 1.07 | 0.94 | 0.82 | 0.69 | 0.48 | 0.39 | 0.26 | 0.24 | 0.16 |
| GSSC-5 h | 1.60 | 1.27 | 0.96 | 0.75 | 0.58 | 0.48 | 0.33 | 0.27 | 0.20 |
| GSSC-10 h | 1.74 | 1.33 | 1.14 | 0.85 | 0.59 | 0.56 | 0.40 | 0.30 | 0.20 |
| GSSC-15 h | 2.62 | 1.80 | 1.34 | 1.06 | 0.75 | 0.57 | 0.43 | 0.29 | 0.22 |

**Table S3.** Figures of merit (FOM) for the MHDP-ChG samples at wavelengths ranging from 710 nm to 870 nm.

| FOM±10% | | | | | | | | | |
| --- | --- | --- | --- | --- | --- | --- | --- | --- | --- |
| S.No | 710nm | 730nm | 750nm | 770nm | 790nm | 810nm | 830nm | 850nm | 870nm |
| precursor glass | 0.25 | 0.25 | 0.31 | 0.47 | 0.41 | 0.54 | 0.63 | 0.57 | 0.61 |
| GSSC-5 h | 0.25 | 0.28 | 0.27 | 0.35 | 0.41 | 0.42 | 0.44 | 0.45 | 0.58 |
| GSSC-10 h | 0.24 | 0.26 | 0.30 | 0.39 | 0.37 | 0.49 | 0.54 | 0.50 | 0.46 |
| GSSC-15 h | 0.30 | 0.28 | 0.27 | 0.37 | 0.38 | 0.35 | 0.47 | 0.34 | 0.42 |

**Table S4.** Optical limiting thresholds (OLT) for the MHDP-ChG samples at wavelengths ranging from 710 nm to 880 nm.

| OLT±10%(μJ/cm^2^) | | | | | |
| --- | --- | --- | --- | --- | --- |
| S.No | 710nm | 750nm | 790nm | 830nm | 880nm |
| precursor glass | 306.8 | 345.0 | 365.1 | 423.3 | 478.4 |
| GSSC-15 h | 80.5 | 129.4 | 306.6 | 400.0 | 466.7 |
